# Stigma, insight and social anxiety in first episode patients with psychosis

**DOI:** 10.1192/j.eurpsy.2021.2140

**Published:** 2021-08-13

**Authors:** M. Efthimiou, P. Petrikis, E. Toki, V. Siafaka, P. Fakitsa, A. Karampas, G. Georgiou, T. Hyphantis

**Affiliations:** 1 Department Of Speech And Language Therapy, UNIVERSITY OF IOANNINA-SCHOOL OF HEALTH SCIENCES, IOANNINA, Greece; 2 Department Of Psychiatry, UNIVERSITY OF IOANNINA-FACULTY OF MEDICINE, IOANNINA, Greece

**Keywords:** Stigma, insight, social anxiety, First episode psychosis

## Abstract

**Introduction:**

People with schizophrenia are considered to be within the most stigmatized social groups. Accurate and efficient detection of stigma and its correlates is essential in patients with psychosis.

**Objectives:**

The purpose of this study was to assess illness insight, stigma, social anxiety and quality of life in patients with a first episode of psychosis and their possible correlations

**Methods:**

The sample of this study consisted of 90 patients with a first episode of psychosis that fulfilled inclusion and exclusion criteria. Tools used for the purpose of this study were Schedule for the assessment of insight-Expanded version, Internalized Stigma for Mental Illness Scale, World Health Organization Quality of Life Assessment - Greek version, Liebowitz Social Anxiety Scale - Greek version. Data were collected and analyzed with SPSS v26.

**Results:**

The study group had good insight (SAI-E score: 20.33±4.449), medium to high stigma values (ISMI score 50.93±7.854), a good enough quality of life (WHOQOL-BREF score: 86.08±10.010) and low levels of social anxiety (LSAS-Gr Fear score: 3.26±8.653; Anxiety score: 2,93±7,596). The results of this study show significant at the 0.01 level 2-tailed correlations as such: (i) a positive and significant relationship between ISMI and LSAS-Gr, (ii) a negative and significant relationship between ISMI and WHOQOL-BREF, and (iii) a negative and moderate relationship between WHOQOL-BREF and LSAS-Gr.

**Conclusions:**

We report medium to high stigma levels, good insight and a good enough quality of life in a sample of first-episode patients with psychosis.

**Disclosure:**

No significant relationships.

